# New 3D Printed Scaffolds Based on Walstromite Synthesized by Sol–Gel Method

**DOI:** 10.3390/jfb15010019

**Published:** 2024-01-08

**Authors:** Ştefania Chiriac, Roxana-Cristina Popescu, Mihnea-Mihăiță Pele, Cristina-Daniela Ghiţulică, Andreia Cucuruz, Ruxandra-Elena Geanaliu-Nicolae, Izabela-Cristina Stancu, Georgeta Voicu, Lucian-Toma Ciocan

**Affiliations:** 1 Department of Science and Engineering of Oxide Materials and Nanomaterials, Faculty of Chemical Engineering and Biotechnologies, National University of Science and Technology Politehnica Bucharest, 011061 Bucharest, Romania; schiriac@imnr.ro (Ş.C.);; 2 National R&D Institute for Non-Ferrous and Rare Metals, INCDMNR-IMNR, 077145 Pantelimon, Romania; 3 Department of Biomaterials and Medical Devices, Faculty of Medical Engineering, National University of Science and Technology Politehnica Bucharest, 011061 Bucharest, Romania; 4 Deparment of Life and Environmental Physics, National Institute for R&D in Physics and Nuclear Engineering-Horia Hulubei, 077125 Magurele, Romania; 5 Advanced Polymer Materials Group, Faculty of Chemical Engineering and Biotechnologies, National University of Science and Technology Politehnica Bucharest, 011061 Bucharest, Romania; 6 Department of Prosthetics Technology and Dental Materials, Carol Davila University of Medicine and Pharmacy, 050474 Bucharest, Romania

**Keywords:** walstromite, bone regeneration, 3D printing, ceramic scaffolds, in vitro bioactivity

## Abstract

This study explores the potential utilization of walstromite (BaCa_2_Si_3_O_9_) as a foundational material for creating new bioceramics in the form of scaffolds through 3D printing technology. To achieve this objective, this study investigates the chemical–mineralogical, morphological, and structural characteristics, as well as the biological properties, of walstromite-based bioceramics. The precursor mixture for walstromite synthesis is prepared through the sol–gel method, utilizing pure reagents. The resulting dried gelatinous precipitate is analyzed through complex thermal analysis, leading to the determination of the optimal calcination temperature. Subsequently, the calcined powder is characterized via X-ray diffraction and scanning electron microscopy, indicating the presence of calcium and barium silicates, as well as monocalcium silicate. This powder is then employed in additive 3D printing, resulting in ceramic scaffolds. The specific ceramic properties of the scaffold, such as apparent density, absorption, open porosity, and compressive strength, are assessed and fall within practical use limits. X-ray diffraction analysis confirms the formation of walstromite as a single phase in the ceramic scaffold. In vitro studies involving immersion in simulated body fluid (SBF) for 7 and 14 days, as well as contact with osteoblast-like cells, reveal the scaffold’s ability to form a phosphate layer on its surface and its biocompatibility. This study concludes that the walstromite-based ceramic scaffold exhibits promising characteristics for potential applications in bone regeneration and tissue engineering.

## 1. Introduction

In various medical fields, such as stomatology and orthopedics, biomaterials designed for bone regeneration play a crucial role. These materials must adhere to a broad spectrum of specifications, including biocompatibility, osseoinduction, controlled bioactivity, an acceptable degradation rate, and appropriate mechanical and physical properties (e.g., density and porosity) [1,2,3].

Over the last decade, there has been a significant surge in the utilization of synthetic biomaterials for the replacement and repair of bone tissue damaged by injury or disease.

The field of bone tissue engineering represents a promising frontier within regenerative medicine, offering potential solutions for the restoration and replacement of damaged or lost bone tissue. Researchers have explored a variety of materials, each possessing distinctive advantages and inherent limitations, shaping the evolving landscape of bone regeneration. Among the most well-known biomaterials studied over time are hydroxyapatite, tricalcium phosphate, bioglass, collagen-based materials, magnesium-based alloys, and polycaprolactone.

Notably, bioceramics based on calcium phosphate (e.g., hydroxyapatite—HAp, β-tricalcium phosphate—β-TCP, HAp/β-TCP) [2] and calcium-silicates (such as bioactive calcium-silicate glasses) have emerged as the most widely employed materials for bone regeneration.

Hydroxyapatite (HA), a bioceramic closely mirroring the mineral composition of natural bone, has attracted attention due to its biocompatibility and osteoconductive properties. Nevertheless, its brittleness poses a challenge for applications demanding robust load-bearing, and its limited resorbability introduces complexities for long-term remodeling [4]. On the other hand, tricalcium phosphate (TCP) displays increased solubility compared to HA, but its mechanical strength may fall short for scenarios requiring substantial load-bearing capabilities [5]. Bioactive glass, with its ability to encourage osteoconduction and stimulate bone formation, presents a promising avenue. However, hurdles arise from its inadequate mechanical strength for load-bearing situations and difficulties in controlling resorption rates [6]. Materials based on collagen, designed to emulate the natural extracellular matrix, provide a biocompatible and biodegradable scaffold for cell attachment. Nevertheless, concerns surrounding their low mechanical strength and rapid degradation draw attention to the potential challenges associated with long-term structural integrity [7]. The exploration of magnesium-based alloys, combining biodegradability with mechanical properties resembling bone, emerges as a novel frontier. However, issues such as rapid corrosion leading to gas production and inflammation highlight the need for refinement in degradation control [8]. Polycaprolactone (PCL), a biodegradable polymer, provides mechanical support but may encounter challenges in aligning degradation rates with new tissue formation, underscoring the ongoing need for materials with enhanced bioactivity and osteoinductivity [9].

As bone tissue engineering continues to progress, the journey towards optimal materials involves delicately balancing biocompatibility, mechanical strength, and controlled degradation. The future of the field hinges on overcoming the limitations of existing materials through innovative research and development, paving the way for transformative approaches for bone regeneration in clinical settings. Thus, in order to avoid these disadvantages, in the scientific field, the emphasis was placed on the exploration of ceramics based on calcium silicate as an alternative to the previously presented biomaterials.

A remarkable feature of calcium-silicate bioceramics is their ability to induce the formation of a layer of hydroxy-carbonate apatite (HAp) on their surface, closely resembling the mineral phase of bone in simulated body fluids (SBF).

Moreover, silicon is recognized as a significant trace element in the human body, with concentrations of approximately 100 ppm in bone and binding to extracellular matrix compounds at concentrations ranging from 200 to 550 ppm. Research has also demonstrated silicon’s presence at the active site of calcification in bone, implicating its direct involvement in the bone mineralization process [2,3,10] From a biological standpoint, calcium is strategically located in the active region of natural bone, where it plays a pivotal role in bone growth and fosters angiogenesis. Additionally, both calcium and silicon have been found to stimulate osteoblast proliferation and differentiation in vitro.

Furthermore, compared to conventional phosphate-based bioceramics, silicate bioceramics offer a broader range of chemical compositions, which can contribute to optimizing various physicochemical properties, including mechanical strength, as well as biological attributes such as bioactivity and degradation [2]. Consequently, in recent years, particular attention has been paid to bioceramics based on materials like wollastonite (CaSiO_3_), akermanite (Ca_2_MgSiO_7_), diopside (CaMgSi_2_O_6_), hardystonite (Ca_2_ZnSi_2_O_7_), bredigite (Ca_7_MgSi_4_O_16_), and merwinite (Ca_3_MgSi_2_O_8_) [10,11,12,13,14]. Furthermore, the literature [3,15,16,17,18,19,20,21,22,23] has highlighted the impact of other cations within these materials on biological behavior. For instance, Zn^2+^ has been shown to promote osteogenesis and mineralization and confer antibacterial properties; Sr^2+^ stimulates bone formation and inhibits bone resorption; and Mg^2+^ supports osteogenesis, mineralization, and angiogenesis.

Recently, significant attention has been directed toward the role of barium in bone repair and regeneration, particularly in the context of osteogenesis [24,25,26]. It has been demonstrated that the incorporation of Ba^2+^ into bioceramic compositions enhances the mineralization of their surfaces under in vitro conditions, as observed during immersion in simulated body fluid (SBF) [26,27,28,29,30]. One such oxide ceramic mineral phase, which includes silicon, calcium, and barium, is known as walstromite. Walstromite is characterized as a cyclosilicate mineral featuring Si_3_O_9_ rings composed of three silicon tetrahedra. Its presence has been reported in the literature in the context of ceramics with electrical properties [31,32,33,34]. Walstromite exhibits a structure similar to wollastonite’s structure (CaSiO_3_), with minimal structural alterations resulting from the substitution of Ca^2+^ with Ba^2+^ [35,36]. Furthermore, it is recognized for its favorable bioactivity from a biological perspective.

In the realm of bone tissue engineering (BTE), the creation of 3D structures to replace, enhance, or regenerate damaged tissue remains a formidable challenge. This challenge is attributed to the diverse requirements these structures must meet, including biocompatibility, rapid production, adequate mechanical properties, irregular shape tailored to the bone defect, and sufficient macroporosity to facilitate the formation of new bone tissue. In essence, the goal of a scaffold designed for bone tissue engineering is to emulate the natural structure of bone. Various conventional methods for obtaining scaffolds in BTE have been reported in the literature over time, such as foaming, sol–gel, polymer sponge, thermal bonding of particles or fibers, and ceramic nanofiber electrospinning techniques. While these methods have demonstrated certain advantages, it has been concluded that achieving an ideal scaffold with proper control over porosity and mechanical strength is nearly impossible with the previously mentioned methods. Issues like irregular pore geometry, inadequate interconnectivity between pores, the use of toxic solvents, low reproducibility, and a prolonged production duration have posed significant challenges [37].

Over the past two decades, the concept of additive manufacturing (AM) has emerged as a flexible and powerful technique for advanced biomedical fabrication. This innovative technology involves the layer-by-layer printing of precise 3D structures based on images obtained through CT or MRI scans using computer-aided design (CAD) techniques. The manufacture of ceramic scaffolds through versatile and reproductible 3D printing techniques represents an increasingly studied and intriguing strategy to circumvent the challenges associated with conventional production methods. Furthermore, this technology efficiently paves the way for the transition of these materials from the research stage to clinical testing. However, the processing of various biomaterials to obtain 3D scaffolds requires the use of different AM technologies based on the characteristics of these materials. In contrast to metallic and polymeric materials, AM techniques for ceramic materials face challenges due to their inherent fragility and high melting points. Additionally, the performance of obtained scaffolds varies depending on the AM technology used [38,39].

In light of these considerations, the present study aimed to investigate the potential utility of walstromite (BaCa_2_Si_3_O_9_) as a foundational material for developing novel bioceramics designed for bone regeneration in the form of scaffolds produced through 3D printing technology. To accomplish this objective, this study examines the chemical–mineralogical, morphological, and structural attributes, as well as the distinctive ceramic and biological properties of walstromite.

## 2. Materials and Methods

### 2.1. Scaffold Synthesis

The sol–gel method was employed to synthesize the precursor mixture required to produce BaCa_2_Si_3_O_9_. This involved using pure reagents, namely tetraethyl orthosilicate (TEOS, Si (OC_2_H_5_)_4_, 99% purity, Sigma-Aldrich, Germany), calcium nitrate tetrahydrate (Ca(NO_3_)_2_·4H_2_O, purity ≥ 99%, Sigma-Aldrich, Germany), and barium acetate (Ba(C_2_H_3_O_2_)_2_, 98.5% purity, Sigma-Aldrich, Germany).

**Figure 1:**
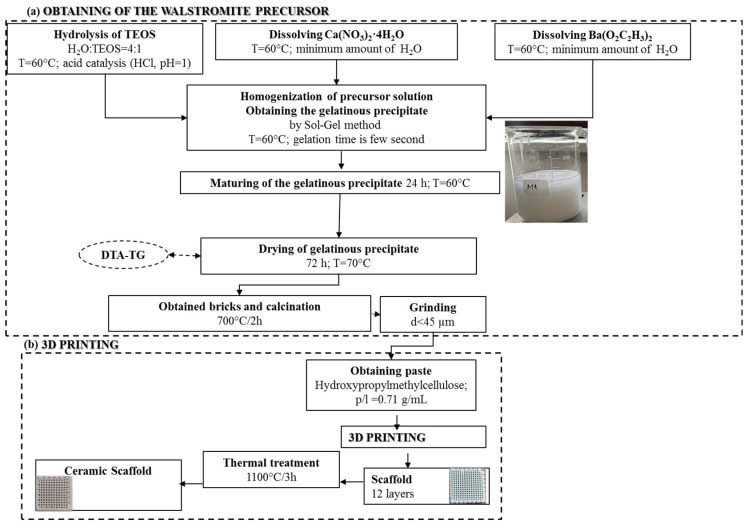
Synthesis scheme of walstromite precursor gel precipitate (**a**) and obtaining ceramic scaffold using 3D printing (**b**).

The process, as illustrated in Figure 1a, began with the solubilization and individual hydrolysis of the precursors, resulting in clear and colorless solutions. These solutions were then homogenized, leading to a rapid gelation process, occurring within seconds. This resulted in the formation of a gelatinous precipitate in a mildly acidic pH range of 5.5–6. The gelatinous precipitate was subsequently subjected to a 72-h period of aging and drying at 60 °C. Following this, it underwent thermal calcination and grinding to yield the precursor powder. This powder was a necessary component to produce a ceramic paste, which was processed using additive 3D printing techniques, like the approach detailed in a previous study [40]. The outcome was a scaffold, as depicted in Figure 1b, which underwent further heat treatment to ultimately yield a ceramic scaffold.

### 2.2. Materials Characterization

#### 2.2.1. Thermal Analysis

The dried gelatinous precipitate was subjected to thermal analysis using Shimadzu DTG-60 equipment (Shimadzu, Kyoto, Japan). This analysis was conducted in an air atmosphere within a temperature range of 20–1000 °C, utilizing a platinum crucible and employing a heating rate of 5 °C per minute. The calcined powder, processed at 700 °C for 2 h, was then evaluated from both compositional and morphological perspectives through X-ray diffraction (XRD) analysis and scanning electron microscopy (SEM), respectively.

#### 2.2.2. XRD Analysis

XRD analyses were conducted using a Shimadzu XRD 6000 diffractometer (Shimadzu, Kyoto, Japan), employing Ni-filtered Cu Kα radiation (λ = 1.5406 Å), with 2 theta values ranging between 5 and 65 degrees. SEM analyses were carried out with an FEI Quanta Inspect F scanning electron microscope (SEM) (FEI, Eindhoven, The Netherlands), equipped with energy dispersive X-ray spectroscopy (EDX). Furthermore, the ceramic scaffold was evaluated regarding its mineralogical phase composition through XRD, and its microstructure was evaluated through SEM analysis.

#### 2.2.3. Ceramic Properties

The ceramic properties, such as apparent density (ρ_a_), absorption (A), and open porosity (P_d_), were determined using the Arthur method [41] and calculated using the following formulas:

$$\rho_{a}=\frac{\left(m_{i}\cdot \rho_{x}\right)}{m_{xa}-m_{x}};\;A=\frac{m_{xa}-m_{i}}{m_{xa}}\cdot 100;\;P_{d}=\frac{m_{xa}-m_{i}}{m_{xa}-m_{x}}\cdot 100,$$

where m_i_—the initial mass of the sample (g); m_xa_—the mass weighed in air of the sample impregnated with xylene (g); m_x_—the mass weighed in xylene of the sample impregnated with xylene (g); ρ_a_—the apparent density (g/cm^3^); A—absorption (%); P_d_—open porosity (%); ρ_x_—the density of xylene (0.86 g/cm^3^).

Compressive strength was assessed using a Shimadzu Autograph AGS-X 20 kN press (Shimadzu, Kyoto, Japan), where force was applied to a scaffold area of 15 × 15 mm, and the specimen’s height was 7 mm. This test was performed on a minimum of three samples.

#### 2.2.4. Biological Evaluation

In vitro bioactivity assessments in simulated body fluid (SBF) were conducted to evaluate the scaffold’s capability to develop a phosphate layer on its surface. To achieve this, the scaffold was immersed in SBF (pH = 7.4) at 37 °C for varying durations (7 and 14 days) within a laboratory water bath. The SBF solution was prepared following the Kokubo protocol [42]. After each immersion period, the scaffold was gently washed with distilled water and allowed to air-dry for approximately 12 h at 60 °C. The scaffold’s mineralization was examined using Fourier-transform infrared spectroscopy (FT-IR) and SEM/EDX.

FT-IR spectra were obtained using a Thermo Scientific Nicolet iS50 spectrometer (Thermo Fisher Scientific, Waltham, MA, USA) in ATR mode (with a diamond crystal).

Additionally, cell viability and cytotoxicity assays (MTT and LDH) were performed to assess the in vitro response to the ceramic scaffold. The cell morphology at the interface with the ceramic scaffold was also evaluated.

*Cell culture:* for cell culture, MG-63 osteoblast-like cells (Cell Line Services CLS, Heidelberg, Germany) were cultivated in Dulbecco’s Modified Eagle Medium (DMEM), supplemented with 10% fetal bovine serum (FBS) and 1% Penicillin-Streptomycin (PAN-Biotech, Aidenbach, Germany), under standard conditions of temperature and humidity (37 °C, 5% CO_2_, 90% humidity).

The ceramic scaffold samples were sterilized using UV exposure for 1 h on each side. Following sterilization, the samples were placed in a 24-well plate (TPP, Trasadingen, Switzerland), and cells were seeded at a concentration of 100,000 cells per 500 µL per well. To create negative and positive controls, cells at the same concentration were directly seeded into the wells of the plate. The samples were incubated for 40 min to allow cell attachment to the surfaces of the scaffolds. Subsequently, the samples were supplemented with an additional 1.5 mL of fresh culture medium and incubated under standard conditions for an additional 48 h. The positive control was treated with 1% Triton-X in complete culture medium.

*Cellular viability measurements (MTT):* The cellular viability of the samples was assessed using the MTT tetrazolium salt viability assay. After the incubation period, the culture medium covering the samples was substituted with 10% MTT solution (prepared from 5 mg/mL MTT in PBS, Serva Electrophoresis, Heidelberg, Germany) in complete culture medium. The mixture was then incubated for 2 h under standard temperature and humidity conditions.

Subsequently, the MTT solution was aspirated, and the resulting formazan crystals were dissolved using dimethyl sulfoxide (DMSO). The quantity of formazan was measured spectrophotometrically at 570 nm. Cell viability was determined with reference to the negative control, which was assigned a value of 100% viability.

*Lactate Dehydrogenase release (LDH):* Following the incubation period, 50 µL of the supernatant from each well was collected and transferred to a 96-well plate to measure the amount of LDH released from the cells after interacting with the biomaterials. To perform this, the CyQUANT LDH Cytotoxicity Assay (Invitrogen, Thermo Fisher Scientific, Waltham, MA, USA) was employed, and the samples were prepared according to the manufacturer’s instructions. The absorbance of each sample was measured spectrophotometrically at 490 nm. The quantity of LDH was determined by comparing the absorbance of each sample to that of the negative control.

The biological investigation data were expressed as mean ± standard deviation (STDEV), and statistical analysis was conducted using the Student *t*-test function.

*Morphological investigations:* To examine the morphology of the osteoblast-like cells cultured on the ceramic samples, confocal microscopy was conducted using the LSM 880 Zeiss microscope (Zeiss, Oberkochen, Germany). To achieve this, the actin filaments of the cells were stained with Alexa Fluor 555 phalloidin (Invitrogen, Thermo Fisher Scientific, Waltham, MA, USA), and the cells’ nuclei were stained with Hoechst 33342 (Invitrogen, Thermo Fisher Scientific, Waltham, MA, USA), following the manufacturer’s instructions.

## 3. Results and Discussion

### 3.1. Gel and Powder Investigation

**Figure 2:**
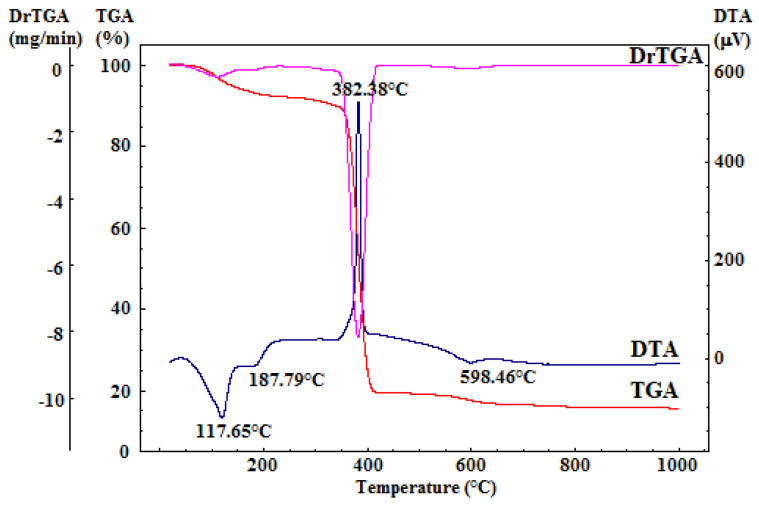
Complex thermal analysis of dried gelatinous precipitate.

The dried gelatinous precipitate underwent comprehensive thermal analysis, as depicted in Figure 2. This analysis provided crucial insights for determining the calcination temperature required for the organic residual component’s combustion and the decomposition of secondary phases (resulting from the reprecipitation of precursor salts or accidental carbonate formation due to exposure to atmospheric CO_2_).

The differential thermal analysis (DTA) curve displays three notable endothermic effects and one exothermic effect, all accompanied by mass loss, as evident in the thermogravimetry (TG) curve. The two endothermic effects occurring within the 50–220 °C range can be attributed to the dehydration of the reprecipitated calcium nitrate, which constitutes a relatively small portion of the mass loss in this temperature range. Additionally, the minor endothermic effect, peaking at around 599 °C, corresponds to the decomposition of the calcium hydroxide formed during synthesis. The prominent exothermic effect, peaking at 382 °C and linked to mass loss, is attributed to the combustion of the residual organic component [43].

Furthermore, the TG curves from the comprehensive thermal analysis reveal that there is no significant mass variation above 700 °C. Consequently, it was determined that calcination of the dried gel precipitate should be conducted at 700 °C, with a two-hour plateau and a heating rate of 1 °C per minute, followed by gradual cooling.

Considering the fine macroscopic nature of the dried gel precipitate, it was uniaxially compressed into cylindrical pellets. This step was taken to prevent material pulverization in the furnace during heat treatment. Following heat treatment, the pellets were milled, and the resulting powder was entirely passed through a 45 µm mesh sieve to attain a finely grained material suitable for 3D printing.

**Figure 3:**
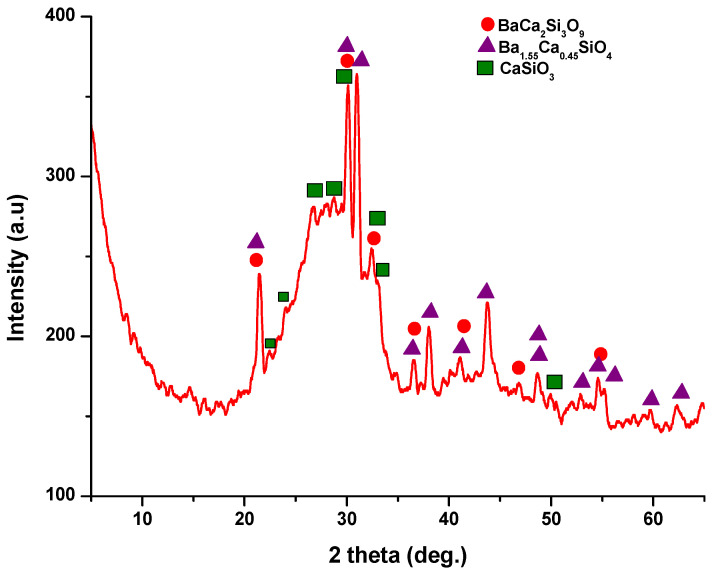
X-ray diffraction pattern for powder calcined at 700 °C/2 h.

The X-ray diffraction (XRD) pattern (Figure 3) of the powder calcined at 700 °C, revealed the presence of distinct peaks corresponding to calcium and barium silicates. Specifically, these peaks correspond to BaCa_2_Si_3_O_9_, as per PDF 015-0063, and Ba_1.55_Ca_0.45_SiO_4_, according to PDF 017-0930. In addition, monocalcium silicate, known as wollastonite (CaSiO_3_), was identified based on PDF 076-0925 and PDF 084-0654. Notably, these peaks are relatively wide and exhibit low intensity, implying the presence of small crystallites.

**Figure 4:**
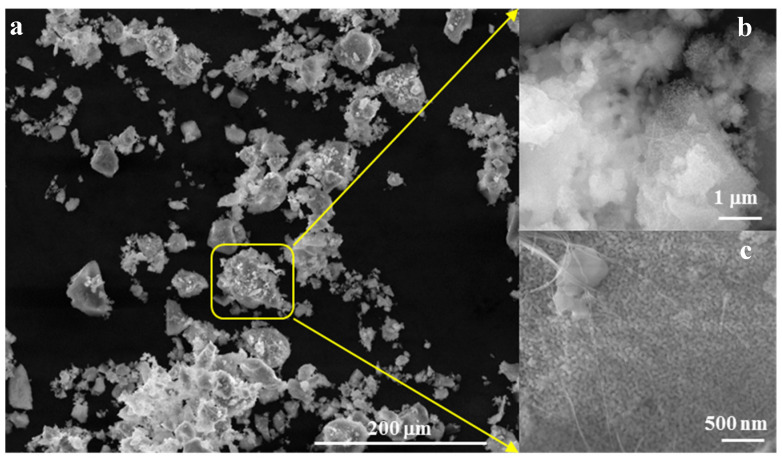
SEM images for powder calcined at 700 °C/2 h (**a**) ×500, (**b**) ×50k, (**c**) ×100k.

Morphologically, as depicted in Figure 4, the powder forms aggregates with diameters below 45 µm (Figure 4a,b), composed of polyhedral particles measuring less than 50 nm and fibrous structures with diameters below 30 nm (Figure 4c).

### 3.2. Walstromit Scaffold—Obtainment and Characterization

**Figure 5:**
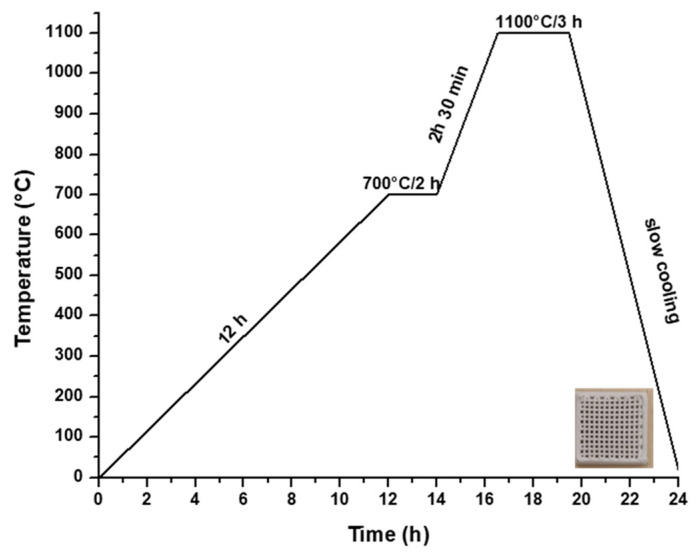
Heat treatment curve applied to obtain the ceramic scaffold.

Hence, to produce a ceramic scaffold using the additive 3D printing method, the utilized material was the powder subjected to heat treatment at 700 °C, in combination with a 15% aqueous solution of hydroxypropyl methyl cellulose (HPMC), as depicted in Figure 1b. The 3D printing process involved the obtainment of homogenous pastes through the combination of powder calcined at 700 °C for 2 h and 15% HPMC, with a powder-to-liquid ratio of 0.71 g/mL. The printing utilized a filament with a thickness of 0.2 mm, encompassing a total of 12 layers. Following the printing, the scaffolds were subjected to a heat treatment at 1100 °C, according to the firing curve illustrated in Figure 5, ultimately yielding ceramic scaffolds denoted as M_02.

The ceramic scaffold obtained was examined with a focus on its specific ceramic properties, as documented in Table 1 (ρ_a_—apparent density, A—absorption, P_d_—open porosity, R_c_—compressive strength), where the open porosity value was assigned to the filament. The strength value confirms the scaffold’s manipulability, and in line with the existing literature, these values fall within the practical use requirements.

**Figure 6:**
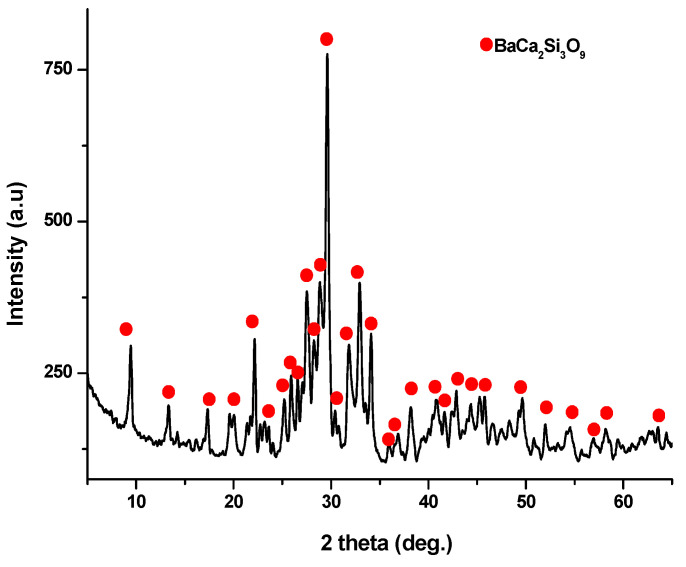
XRD pattern for ceramic scaffold obtained at 1100 °C/3 h.

X-ray diffraction analysis (as depicted in Figure 6) conducted on the ceramic scaffold, which underwent heat treatment at 1100 °C for 3 h, revealed the formation of walstromite as a single phase, as indicated by PDF 015-0063 and PDF 073-1907.

**Figure 7:**
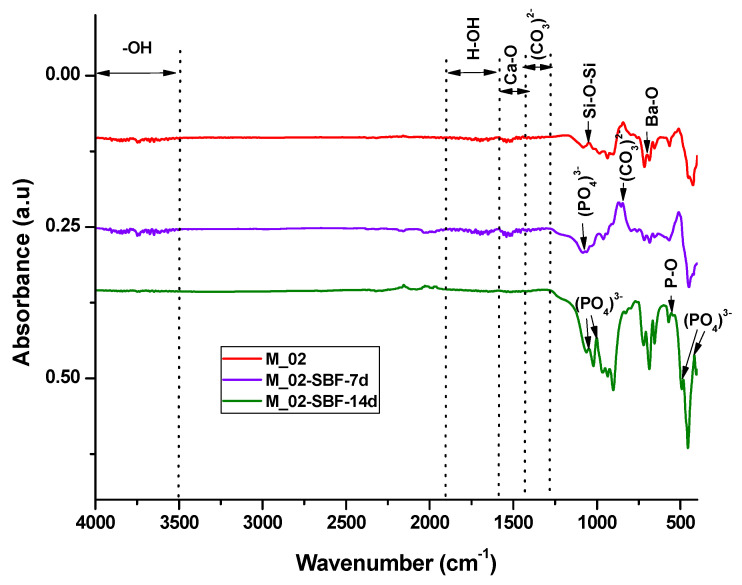
FTIR spectra of unimmersed and immersed M_02 scaffolds in SBF for 7 and 14 days, respectively.

**Figure 8:**
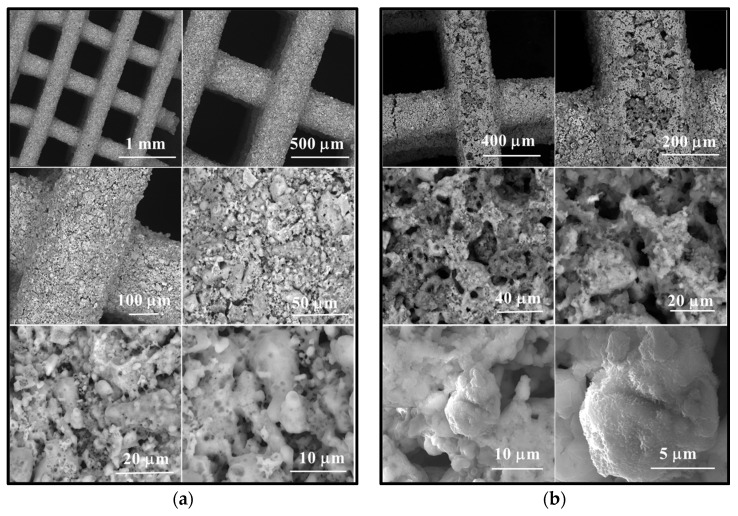
Scanning electron microscopy images of scaffold M_02 (**a**) unimmersed in SBF and (**b**) immersed in SBF for 14 days, respectively.

**Figure 9:**
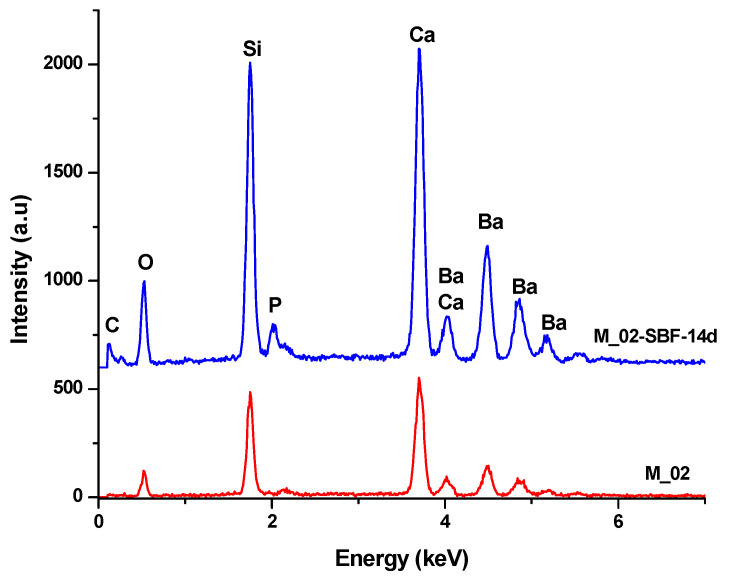
EDX spectra of unimmersed (M_02) and 14-day SBF-immersed scaffolds (M_02-SBF-14d).

The in vitro behavior of the M_02 ceramic scaffold was investigated through immersion in simulated body fluid (SBF) for 7 and 14 days, as well as through interaction with cells. The sample immersed in SBF was characterized using Fourier-transform infrared (FTIR) spectrometry (see Figure 7 and Table 2) and scanning electron microscopy (refer to Figure 8), which was coupled with energy-dispersive X-ray (EDX) analysis (see Figure 9).

The infrared spectroscopy data, as depicted in Figure 7 and summarized in Table 2, reveal characteristic absorption bands of Ba-O (at 700 cm^−1^), Si-O-Si (at 1043 and 923–1100 cm^−1^), and Ca-O (approximately 1460 cm^−1^) for the unimmersed scaffold. These bands exhibit a decrease in intensity or even disappear upon immersion in simulated body fluid (SBF), indicating an interaction between the scaffold and the fluid.

For the samples immersed in SBF, absorption band characteristics for partially carbonated hydroxyapatite become evident: bands associated with PO_4_^3−^ (at 551, 1073, 480, and 414 cm^−1^), -OH (ranging from 3500 to 4000 cm^−1^), and CO_3_^2−^ (at 855 and 1338–1400 cm^−1^). It is noteworthy that the intensity of these absorption bands increases with prolonged immersion, suggesting a higher amount of phosphate phase formation upon contact with SBF [44,45].

The scanning electron microscopy (SEM) images presented in Figure 8 reveal the following observations:

- In the case of the unimmersed ceramic scaffold (M_02), the filaments appear continuously, exhibit uniform thickness, and display open porosity. This information is consistent with the data in Table 1. Additionally, both intra- and inter-granular porosity is observed, suggesting enhanced circulation of physiological fluids and growth factors within the material at the implantation site.
- For the sample immersed for 14 days, SEM images clearly illustrate alterations in the morpho-structural and surface characteristics of the scaffold filaments. Notably, the surface roughness of the filaments increases, indicating an interaction between the scaffold and simulated body fluid (SBF). At higher magnifications, quasi-spherical particles composed of very fine plates can be observed. These particles are attributed to the partially carbonate apatite phase formed during surface mineralization, which aligns with the FTIR spectroscopy findings mentioned earlier.
- Furthermore, the presence of phosphorus in the EDX analysis, as shown in Figure 9, confirms the interaction between SBF and the material. It is worth noting that the SBF solution, in which the scaffold was immersed for 14 days, has a slightly basic pH value of 8.5.

**Figure 10:**
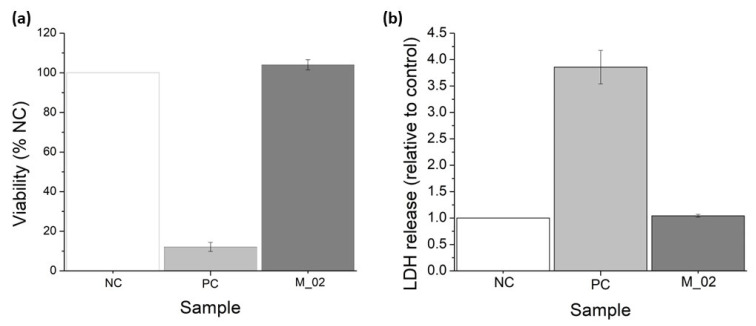
Viability assessment of the 3D-printed ceramic samples: (**a**) MTT tetrazolium-salt viability assay; (**b**) lactate dehydrogenase (LDH) release for MG-63 osteoblast-like cells cultured in the presence of M_02 samples during 48 h.

MTT and LDH assays were conducted to assess the in vitro performance of M_02 using MG-63 osteoblast-like cells, as depicted in Figure 10. The MTT tetrazolium salt viability assay serves as an indirect method for evaluating cell viability. This approach measures the overall metabolic activity of osteoblast-like cells after 48 h of incubation with 3D-printed ceramic samples. The cells’ ability to metabolize MTT into formazan is directly proportional to their viability. Consequently, osteoblast-like cells cultured in the presence of M_02 samples exhibited a biocompatible behavior like the negative control (NC), with a determined cell viability of 104.07 ± 2.56% (*p* = 0.11). In contrast, the determined viability for the positive control (PC) was significantly lower at 12.12 ± 2.26 (*p* < 0.001), as shown in Figure 10a.

On the other hand, the measurement of lactate dehydrogenase (LDH) release into the extracellular medium serves as an indicator of cell death resulting from membrane ruptures. LDH is a cytosolic enzyme, and its presence outside the cell is an indicator of necrosis. In the case of the 3D-printed ceramic samples, there were no significant quantities of released LDH detected, with a value of 1.04 ± 0.02 (*p* = 0.09), compared to the negative control (see Figure 10b). Conversely, the positive control samples showed a higher LDH release of 3.85 ± 0.31 (*p* = 0.004).

**Figure 11:**
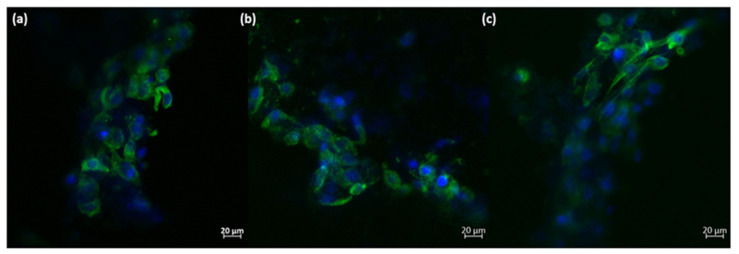
Morphology of the MG-63 osteoblast-like cells cultured onto 3D-printed M_02 ceramic samples; green—actin filaments; blue—nuclei (subfigures (**a**–**c**): different zoomed areas).

Based on these quantitative measurements, it can be concluded that the presence of the 3D-printed ceramic samples did not significantly affect the viability of osteoblast-like cells. Additionally, a minor increase in cell proliferation can be observed, although it is not statistically significant compared to the negative control. The morphology of the osteoblast-like cells cultured on the 3D-printed ceramic samples appears normal, with cells maintaining a characteristic prolonged polygonal shape, typical of osteoblast cells (as shown in Figure 11). Furthermore, the actin filaments of MG-63 cells closely follow the scaffold’s architecture, indicating that the M_02 samples provide a suitable substrate for the attachment and growth of osteoblast-like cells.

The main purpose of scaffolds in bone tissue engineering is to support the cells’ attachment and growth, enabling a 3D biomimetic environment. Here, the obtained 3D walstromite scaffolds clearly provide a suitable architecture for the osteoblast-like cells, comparable with that of the natural bone. Moreover, the rough surface of the filaments facilitates the adherence of the MG-63 cells, which follow the conformation of the 3D structure.

The hydroxyapatite phase that occurred following the immersion in the simulated body fluid enhances the texture of the 3D scaffold, resulting in round-shaped calcium phosphate aggregates lining the exposed extremities of the material. These structures on their own showcase a high specific surface area due to the platelet-like structure, providing many attachment points for the osteoblast cells. Due to these morphological features, the cells are able to form strong connection to the scaffold, finally leading to improved osseointegration into the in vivo environment. The first biological molecules interacting with the biomaterial upon immersion in a biological environment are the proteins in the media. Then, the attachment of the cells happens. It has been shown that the nanotopography of the calcium phosphate-based materials influence the conformation of these proteins, leading to an improved response of the osteoblast cells when they finally adhere to the material [46]. Additionally, it has been shown that bioactive materials leading to the formation of nanostructured hydroxyapatite promote the cells’ proliferation and differentiation in vitro and in vivo [47].

Additionally, the proposed new material offers the biocompatible setting required for the growth of the osteoblast cells, which is a mandatory requirement of biomaterials, proving a behavior comparable to that of classic hydroxyapatite-based [38,48,49] or calcium silicate-based [50,51] scaffolds obtained through 3D printing. To the best of our knowledge, this is the first time the walstromite biocompatibility was investigated for osteoblast-like cells in tissue engineering applications. Moreover, the proposed walstromite-based 3D-printed scaffolds clearly offer prospective clinical applications in the management of bone defects, such as limited defects, defects where bone fragments have contact, and segmental defects. The large applicability is given by the implication of the 3D printing technique and possibility to employ a previous CAD-CAM scanning step in order to design the scaffold according to the patient’s need. This opens up future implications of the proposed devices in the personalized medical management of bone defect reconstruction.

## 4. Conclusions

This study has explored the potential utility of walstromite (BaCa_2_Si_3_O_9_) as a fundamental material for the development of novel bioceramics for bone regeneration using 3D printing technology.

The research rigorously characterizes the synthesized bioceramic scaffold through multiple techniques, including X-ray diffraction (XRD) and scanning electron microscopy (SEM). These analyses provide essential insights into the material’s composition and morphology. This study confirms that the specific ceramic properties of the scaffold, including apparent density, absorption, open porosity, and compressive strength, fall within the acceptable range for practical utilization in bone regeneration.

Through immersion in simulated body fluid (SBF), this study demonstrates the scaffold’s capacity to form a phosphate layer on its surface, indicating a high degree of in-vitro bioactivity, a critical attribute for bone tissue engineering. The assessment of the ceramic scaffold’s biocompatibility using MG-63 osteoblast-like cells underscores its suitability as a platform for cell growth and reveals no cytotoxic effects. Morphological analysis of the cultured cells on the ceramic scaffold shows that the material accommodates the attachment and growth of osteoblast-like cells.

In summary, this investigation provides substantial evidence supporting the viability of walstromite-based bioceramics, particularly when fabricated as 3D-printed scaffolds, for applications in bone tissue regeneration. This research opens avenues for further exploration and development in the realm of medical biomaterials and bone regenerative therapies.

## Figures and Tables

**Table 1:** Specific ceramic properties for ceramic scaffold with 0.2 mm filament thickness (arithmetic mean of values determined based on three samples).

Sample	ρ_a_ (g/cm^3^)	A (%)	P_d_ (%)	R_c_ (MPa)
M_02	2.67 ± 0.03	12.05 ± 0.03	41.34 ± 0.03	2.56 ± 0.03

**Table 2:** Centralization of FTIR absorption bands for unimmersed and immersed M1_02 scaffold for 7 and 14 days.

Unimmersed in SBF		Immersed in SBF:			
		7 Days		14 Days	
Wavenumber (cm^−1^)	Functional Group	Wavenumber (cm^−1^)	Functional Group	Wavenumber (cm^−1^)	Functional Group
700	Ba-O	855	C-O din CO_3_^2−^	480/414	(PO_4_)^3−^
1043	Si-O-Si	1073	PO_4_^3−^	551	P-O from (PO_4_)^3−^
923–1100	[SiO_4_]^4−^	1338–1400	CO_3_^2−^	855	C-O from CO_3_^2−^
1460	Ca-O	1460	Ca-O	1052/1000	PO_4_^3−^
		1600–1900		atmosphere water	
		3500–4000		–OH from hydroxyapatite	

## Data Availability

Data are contained within the article.

## References

[1] Maksoud F.J., Velázquez de la Paz M.F., Hann A.J., Thanarak J., Reilly G.C., Claeyssens F., Green N.H., Zhang Y.S. (2022). Porous Biomaterials for Tissue Engineering: A Review. J. Mater. Chem. B.

[2] Youness R.A., Tag El-deen D.M., Taha M.A. (2023). A Review on Calcium Silicate Ceramics: Properties, Limitations, and Solutions for Their Use in Biomedical Applications. Silicon.

[3] Gu X., Li Y., Qi C., Cai K. (2022). Biodegradable Magnesium Phosphates in Biomedical Applications. J. Mater. Chem. B.

[4] Bose S., Roy M., Bandyopadhyay A. (2012). Recent Advances in Bone Tissue Engineering Scaffolds. Trends Biotechnol..

[5] Guo B., Lei B., Li P., Ma P.X. (2015). Functionalized Scaffolds to Enhance Tissue Regeneration. Regen. Biomater..

[6] Hoppe A., Güldal N.S., Boccaccini A.R. (2011). A Review of the Biological Response to Ionic Dissolution Products from Bioactive Glasses and Glass-Ceramics. Biomaterials.

[7] Murphy C.M., Haugh M.G., O’Brien F.J. (2010). The Effect of Mean Pore Size on Cell Attachment, Proliferation and Migration in Collagen–Glycosaminoglycan Scaffolds for Bone Tissue Engineering. Biomaterials.

[8] Witte F., Kaese V., Haferkamp H., Switzer E., Meyer-Lindenberg A., Wirth C.J., Windhagen H. (2005). In Vivo Corrosion of Four Magnesium Alloys and the Associated Bone Response. Biomaterials.

[9] John C., Middleton A.J.T. (2000). Synthetic Biodegradable Polymers as Orthopedic Devices. Biomaterials.

[10] Yu Q., Chang J., Wu C. (2019). Silicate Bioceramics: From Soft Tissue Regeneration to Tumor Therapy. J. Mater. Chem. B.

[11] Wu C., Chang J. (2013). A Review of Bioactive Silicate Ceramics. Biomed. Mater..

[12] Gao C., Peng S., Feng P., Shuai C. (2017). Bone Biomaterials and Interactions with Stem Cells. Bone Res..

[13] Mohammadi H., Sepantafar M., Ostadrahimi A. (2015). The Role of Bioinorganics in Improving the Mechanical Properties of Silicate Ceramics as Bone Regenerative Materials. J. Ceram. Sci. Technol..

[14] Nicoara A.I., Alecu A.E., Balaceanu G.-C., Puscasu E.M., Vasile B.S., Trusca R. (2023). Fabrication and Characterization of Porous Diopside/Akermanite Ceramics with Prospective Tissue Engineering Applications. Materials.

[15] Lin K., Xia L., Li H., Jiang X., Pan H., Xu Y., Lu W.W., Zhang Z., Chang J. (2013). Enhanced Osteoporotic Bone Regeneration by Strontium-Substituted Calcium Silicate Bioactive Ceramics. Biomaterials.

[16] Chasapis C.T., Loutsidou A.C., Spiliopoulou C.A., Stefanidou M.E. (2012). Zinc and Human Health: An Update. Arch. Toxicol..

[17] Deng L., Huang L., Pan H., Zhang Q., Que Y., Fan C., Chang J., Ni S., Yang C. (2023). 3D Printed Strontium–Zinc-Phosphate Bioceramic Scaffolds with Multiple Biological Functions for Bone Tissue Regeneration. J. Mater. Chem. B.

[18] Shao H., Liu A., Ke X., Sun M., He Y., Yang X., Fu J., Zhang L., Yang G., Liu Y. (2017). 3D Robocasting Magnesium-Doped Wollastonite/TCP Bioceramic Scaffolds with Improved Bone Regeneration Capacity in Critical Sized Calvarial Defects. J. Mater. Chem. B.

[19] Ghițulică C.-D., Cucuruz A., Voicu G., Cucuruz A.T., Dinescu S., Selaru A., Costache M. (2019). Ceramics Based on Calcium Phosphates Substituted with Magnesium Ions for Bone Regeneration. Int. J. Appl. Ceram. Technol..

[20] He F., Yuan X., Lu T., Wang Y., Feng S., Shi X., Wang L., Ye J., Yang H. (2022). Preparation and Characterization of Novel Lithium Magnesium Phosphate Bioceramic Scaffolds Facilitating Bone Generation. J. Mater. Chem. B.

[21] O’Neill E., Awale G., Daneshmandi L., Umerah O., Lo K.W.-H. (2018). The Roles of Ions on Bone Regeneration. Drug Discov. Today.

[22] Mao L., Xia L., Chang J., Liu J., Jiang L., Wu C., Fang B. (2017). The Synergistic Effects of Sr and Si Bioactive Ions on Osteogenesis, Osteoclastogenesis and Angiogenesis for Osteoporotic Bone Regeneration. Acta Biomater..

[23] Chen Z., Zhang W., Wang M., Backman L.J., Chen J. (2022). Effects of Zinc, Magnesium, and Iron Ions on Bone Tissue Engineering. ACS Biomater. Sci. Eng..

[24] Kovrlija I., Locs J., Loca D. (2021). Incorporation of Barium Ions into Biomaterials: Dangerous Liaison or Potential Revolution?. Materials.

[25] Singh J., Kumar V., Vermani Y.K., Al-Buriahi M.S., Alzahrani J.S., Singh T. (2021). Fabrication and Characterization of Barium Based Bioactive Glasses in Terms of Physical, Structural, Mechanical and Radiation Shielding Properties. Ceram. Int..

[26] Arepalli S.K., Tripathi H., Vyas V.K., Jain S., Suman S.K., Pyare R., Singh S.P. (2015). Influence of Barium Substitution on Bioactivity, Thermal and Physico-Mechanical Properties of Bioactive Glass. Mater. Sci. Eng. C.

[27] Mabrouk M., Ibrahim Fouad G., Beherei H.H., Das D.B. (2022). Barium Oxide Doped Magnesium Silicate Nanopowders for Bone Fracture Healing: Preparation, Characterization, Antibacterial and In Vivo Animal Studies. Pharmaceutics.

[28] Myat-Htun M., Mohd Noor A.-F., Kawashita M., Baba Ismail Y.M. (2020). Enhanced Sinterability and in Vitro Bioactivity of Barium-Doped Akermanite Ceramic. Ceram. Int..

[29] Yazdanpanah A., Moztarzadeh F. (2019). Synthesis and Characterization of Barium–Iron Containing Magnetic Bioactive Glasses: The Effect of Magnetic Component on Structure and in Vitro Bioactivity. Colloids Surf. B Biointerfaces.

[30] Chiu Y.-C., Lin Y.-H., Chen Y.-W., Kuo T.-Y., Shie M.-Y. (2023). Additive Manufacturing of Barium-Doped Calcium Silicate/Poly-ε-Caprolactone Scaffolds to Activate CaSR and AKT Signalling and Osteogenic Differentiation of Mesenchymal Stem Cells. J. Mater. Chem. B.

[31] Wisniewski W., Thieme C., Müller R., Reinsch S., Groß-Barsnick S.-M., Rüssel C. (2018). Oriented Surface Nucleation and Crystal Growth in a 18BaO·22CaO·60SiO_2_ Mol% Glass Used for SOFC Seals. CrystEngComm.

[32] Yang J., Xie Q., Ao L., Wu S., Zhu X., Zhong Q., Xu Y., Fang Z., Tang X., Tang B. (2023). Structure and Microwave Dielectric Properties of Novel Walstromite-Type MCa_2_Si_3_O_9_ (M = Ba, Sr) Ceramics. Ceram. Int..

[33] Müller M., Jüstel T. (2015). Energy Transfer and Unusual Decay Behaviour of BaCa_2_Si_3_O_9_: Eu^2+^, Mn^2+^ Phosphor. Dalton Trans..

[34] Rangel-Hernández V.H., Fang Q., Babelot C., Lohoff R., Blum L. (2020). An Experimental Investigation of Fracture Processes in Glass-Ceramic Sealant by Means of Acoustic Emission. Int. J. Hydrogen Energy.

[35] Raut S.K., Dhoble N.S., Park K., Dhoble S.J. (2014). Precipitation Based Synthesis and Luminescence of Ln^3+^ (Eu, Ce, Dy, Sm, Tb) Activated BaCa_2_Si_3_O_9_-Walstromite Cyclosilicate Phosphors. Mater. Chem. Phys..

[36] Barkley M.C., Downs R.T., Yang H. (2011). Structure of Walstromite, BaCa_2_Si_3_O_9_, and Its Relationship to CaSiO_3_-Walstromite and Wollastonite-II. Am. Mineral..

[37] Monfared M.H., Nemati A., Loghman F., Ghasemian M., Farzin A., Beheshtizadeh N., Azami M. (2022). A Deep Insight into the Preparation of Ceramic Bone Scaffolds Utilizing Robocasting Technique. Ceram. Int..

[38] Thurzo A., Gálfiová P., Nováková Z.V., Polák Š., Varga I., Strunga M., Urban R., Surovková J., Leško Ľ., Hajdúchová Z. (2022). Fabrication and In Vitro Characterization of Novel Hydroxyapatite Scaffolds 3D Printed Using Polyvinyl Alcohol as a Thermoplastic Binder. Int. J. Mol. Sci..

[39] Qu H. (2020). Additive Manufacturing for Bone Tissue Engineering Scaffolds. Mater. Today Commun..

[40] Dobriţa C.-I., Bădănoiu A.-I., Voicu G., Nicoară A.-I., Dumitru S.-M., Puşcaşu M.-E., Chiriac Ș., Ene R., Iordache F. (2023). Porous Bioceramic Scaffolds Based on Akermanite Obtained by 3D Printing for Bone Tissue Engineering. Ceram. Int..

[41] (1995). Ceramic Tiles–Part 3. Determination of Water Absorption, Apparent Porosity, Apparent Relative Density and Bulk Density.

[42] Kokubo T., Kim H.-M., Kawashita M. (2003). Novel Bioactive Materials with Different Mechanical Properties. Biomaterials.

[43] Zhao A., Xiong B., Han Y., Tong H. (2022). Thermal Decomposition Paths of Calcium Nitrate Tetrahydrate and Calcium Nitrite. Thermochim. Acta.

[44] Krzątała A., Krüger B., Galuskina I., Vapnik Y., Galuskin E. (2020). Walstromite, BaCa_2_(Si_3_O_9_), from Rankinite Paralava within Gehlenite Hornfels of the Hatrurim Basin, Negev Desert, Israel. Minerals.

[45] Ansari M.A., Jahan N. (2021). Structural and Optical Properties of BaO Nanoparticles Synthesized by Facile Co-Precipitation Method. Mater. Highlights.

[46] Thamma U., Kowal T.J., Falk M.M., Jain H. (2021). Nanostructure of Bioactive Glass Affects Bone Cell Attachment via Protein Restructuring upon Adsorption. Sci. Rep..

[47] Williams D.F. (2022). Biocompatibility Pathways and Mechanisms for Bioactive Materials: The Bioactivity Zone. Bioact. Mater..

[48] Kim Y., Lee E.-J., Davydov A.V., Frukhtbeyen S., Seppala J.E., Takagi S., Chow L., Alimperti S. (2021). Biofabrication of 3D Printed Hydroxyapatite Composite Scaffolds for Bone Regeneration. Biomed. Mater..

[49] Liu S., Hu Y., Zhang J., Bao S., Xian L., Dong X., Zheng W., Li Y., Gao H., Zhou W. (2019). Bioactive and Biocompatible Macroporous Scaffolds with Tunable Performances Prepared Based on 3D Printing of the Pre-Crosslinked Sodium Alginate/Hydroxyapatite Hydrogel Ink. Macromol. Mater. Eng..

[50] Dai K., Yang Z., Ding L., Yang Z., Hang F., Cao X., Chen D., Zhao F., Chen X. (2023). 3D-Printed Strontium-Doped BG-CaSiO_3_-HA Composite Scaffolds Promote Critical Bone Defect Repair by Improving Mechanical Strength and Increasing Osteogenic Activity. Ceram. Int..

[51] Yang C., Wang X., Ma B., Zhu H., Huan Z., Ma N., Wu C., Chang J. (2017). 3D-Printed Bioactive Ca_3_SiO_5_ Bone Cement Scaffolds with Nano Surface Structure for Bone Regeneration. ACS Appl. Mater. Interfaces.

